# Simulation and Algorithmic Optimization of the Cutting Process for the Green Machining of PM Green Compacts

**DOI:** 10.3390/ma17163963

**Published:** 2024-08-09

**Authors:** Yuchen Zhang, Dayong Yang, Lingxin Zeng, Zhiyang Zhang, Shuping Li

**Affiliations:** School of Mechanical and Automotive Engineering, Guangxi University of Science and Technology, Liuzhou 545006, China

**Keywords:** algorithm optimization, powder metallurgy, finite element, green compacts

## Abstract

Powder metallurgy (PM) technology is extensively employed in the manufacturing sector, yet its processing presents numerous challenges. To alleviate these difficulties, green machining of PM green compacts has emerged as an effective approach. The aim of this research is to explore the deformation features of green compacts and assess the impact of various machining parameters on the force of cutting. The cutting variables for compacts of PM green were modeled, and the cutting process was analyzed using Abaqus (2022) software. Subsequently, the orthogonal test ANOVA method was utilized to evaluate the significance of each parameter for the cutting force. Optimization of the machining parameters was then achieved through a genetic algorithm for neural network optimization. The investigation revealed that PM green compacts, which are brittle, undergo a plastic deformation stage during cutting and deviate from the traditional model for brittle materials. The findings indicate that cutting thickness exerts the most substantial influence on the cutting force, whereas the speed of cutting, the tool rake angle, and the radius of the rounded edge exert minimal influence. The optimal parameter combination for the cutting of PM green compacts was determined via a genetic algorithm for neural network optimization, yielding a cutting force of 174.998 N at a cutting thickness of 0.15 mm, a cutting speed of 20 m/min, a tool rake angle of 10°, and a radius of the rounded edge of 25 μm, with a discrepancy of 4.05% from the actual measurement.

## 1. Introduction

Conventional machining is almost always performed by removing excess material to ensure that the workpiece meets machining standards [1,2]; however, this type of machining wastes a large amount of material, with such waste being inconsistent with the main idea of green machinery manufacturing [3]. Powder metallurgy (PM) is used to manufacture metal parts by extruding metal powder to form green compacts and then sintering them, which is more material-efficient than traditional machining [4] and has been applied in many manufacturing fields [5,6,7,8,9]. However, it is almost impossible to process PM products in a single mold, and they require a small amount of processing [10].

The processing of PM products post-sintering presents substantial challenges, which inhibits the advancement of the powder metallurgy field [11,12]. Further innovation in the powder metallurgy (PM) industry has been hindered by several challenges. Ceramic machining shares similarities with PM machining; both processes involve the use of green compacts, which are subsequently sintered. However, after sintering, ceramic green compacts become difficult to process [13]. To address this issue in the ceramics industry, green compacts are processed before sintering [14]. This technique offers valuable insights for the powder metallurgy field [15] and suggests a potential strategy to alleviate the processing difficulties encountered in PM [16]. Green machining has become crucial in powder metallurgy material preparation, with the aim of achieving precise geometric features through the machining of green compacts before sintering and before addressing the challenges associated with machining these materials [17,18,19]. Paradis et al. [20] investigated the cold sintering process for surface-modified iron particles and revealed that this approach encourages the formation of a co-continuous phosphate phase among iron powder particles, which significantly improves the strength and density of the green compacts. These attained relative densities that reached 95%, and the transverse fracture strength was approximately 75 MPa, nearly sixfold greater than that of traditional powdered metal iron green compacts. This advancement underscores the growing academic interest in the green machining of PM green compacts, spotlighting it as a pivotal area of research.

While investigating the green compact machining mechanism, Robert-Perron et al. [21] observed that the tensile properties of parts machined in the green state were akin to those of parts machined post-sintering, as evidenced by experiments on cylindrical PM green compact sintering performance. This finding suggests that pre-sintering does not detrimentally impact the material properties. Moreover, Yang et al. [22] have elucidated unique material removal techniques such as particle shear deformation, stripping, and plowing/extrusion through geometric modeling of the green machining process of PM. The warm mold compaction method was shown to significantly decrease the porosity during the powder metallurgy process. Shi et al. [23] have explored the impact of warm mold compaction on the mechanical properties of iron-based powder metallurgy sintering and revealed that specimens prepared via warm mold compaction exhibited increased hardness, tensile strength, and yield strength.

Complementing the detailed studies on green machining mechanisms, extensive research has been conducted on machined surface quality, surface roughness minimization, and multi-objective optimization. For instance, according to research by Kulkarni Harshal et al. [24], the quality of machined surfaces improves with decreasing feed rate. Moreover, Goncalves et al. [25] observed from turning tests that increasing the radius of the rounded edge reduces surface roughness. Moreover, Kumar et al. [26] utilized the Gray–Taguchi approach for multiple objectives in the optimization of aerospace-grade alloys constructed of titanium, showing, through statistical assessment, that the speed of cutting substantially affects the quality of the machined surface.

Although extensive research has been conducted on various aspects of machining, the impact of cutting force has not been thoroughly explored. Although most researchers believe that the cutting force is small [16], due to the low strength of powder metallurgy green compacts, the workpieces are susceptible to damage by the cutting force during machining [27]. Hence, a systematic analysis of the way different machining parameters influence the cutting force is vital for a deeper understanding of the cutting characteristics of materials.

Despite the paucity of research on cutting forces within the realm of PM green machining utilizing optimization algorithms, employing these algorithms for cutting force optimization could provide a detailed understanding of how different machining parameters interact with the force of cutting. This knowledge is crucial for enhancing the quality and productivity of machined parts.

Therefore, this study sets out to achieve the following objectives:

1. Investigate deformation characteristics—to investigate the deformation characteristics of PM green compacts during the cutting process and evaluate the influence of various machining parameters on cutting forces.
2. Develop a simulation model—to develop a cutting process model for PM green compacts using Abaqus (2022) software for simulation.
3. Assess parameter significance—to use orthogonal test ANOVA methods to assess the significance of different machining parameters on cutting forces.
4. Optimize machining parameters—to optimize machining parameters through the application of a genetic algorithm for neural network optimization.
5. Validate the model—to validate the developed cutting model with experimental procedures.
6. Analyze cutting force variations—to analyze the variations in cutting forces under different machining parameters to determine the optimal cutting conditions.

By addressing these objectives, this research aims to contribute to a deeper understanding of PM green machining and to propose effective strategies for optimizing the cutting process.

## 2. Finite Element Modeling

### 2.1. Microstructure of Materials

**Figure 1:**
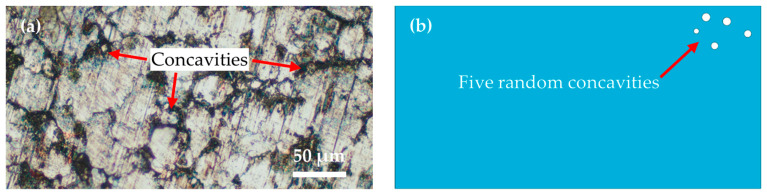
Microscopic morphology of the PM green compacts and modeling of the workpieces. (**a**) Microscopic topography of the PM green compacts. (**b**) Model of the workpiece.

When examining the various preparation processes for green PM compact materials, it was observed that the material porosity of powder metallurgy green compacts produced through different pressing techniques exhibited variability [28]. The microstructural morphology of the PM green compact materials is depicted in Figure 1a, revealing an approximate porosity of 12% within the material. For modeling purposes, Abaqus was employed, and, to alleviate the computational demands of computer simulation, a singular microstructure representative of the pore structure in PM green compacts was addressed. By arbitrarily placing five pore structures on the workpiece model substrate, the simulation was confined to the upper right quadrant of the model, as illustrated in Figure 1b.

### 2.2. Model Parameters

In the simulation, the workpiece was treated as a plastic body, and the tool was treated as an analytically rigid entity. The material parameters were established through a literature review and information assessment from tests on PM green compact materials. This research involved conducting tensile, strength, and compressive assessments on green compact test samples, and each test was performed six times to ascertain the reliability of the results and to calculate average values. The data compiled from these tests are organized in Table 1, which presents the pertinent performance parameters for PM–green compact materials. Table 2 shows the chemical composition of the green compact material.

To facilitate a more intuitive understanding of the material removal process in simulated PM green compacts during cutting and to assist in the analysis of relevant experimental parameters, the initial parameters for the tool model were a rounded edge radius of 10 μm, an angle for the tool rake of 10°, and a clearance angle of 20°. The selection of a carbide-coated tool as the tool material was based on parameters provided by the tool manufacturer. The feed rate in this study is constant at 0.2 mm/r.

**Figure 2:**
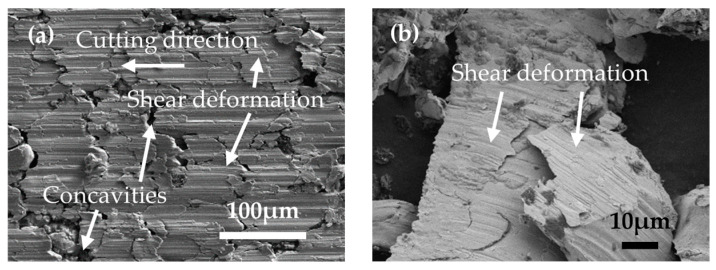
Machined surfaces and chips observed with an electron microscope. (**a**) Machined surfaces. (**b**) Chips.

Figure 2a illustrates the machined surface of the specimen as captured by an electron microscope, revealing that the primary mode of material removal from the PM green compact specimen during cutting is predominantly plastic deformation. Similarly, Figure 2b displays images of the chips that underwent plastic deformation, which were also observed under an electron microscope. Consequently, this study adopts the Johnson–Cook model [29], a model extensively applied to the task of delineating the strength thresholds and failure mechanisms of metallic materials under conditions of significant strains and high strain rates, which are notably prevalent in simulations of metal cutting [30]. The parameters for the Johnson–Cook constitutive model applicable to PM green compact materials are detailed in Table 3 and are derived from experimental data that were subsequently fitted to the primary data and refined through modeling.

### 2.3. Meshing and Assembly

**Figure 3:**
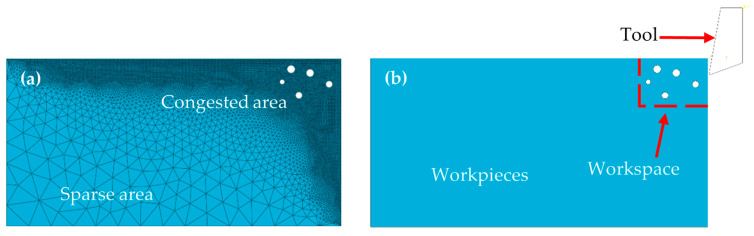
Diagrams of model meshing and assembly. (**a**) Mesh distribution. (**b**) Overall assembly.

In this study, the contact between the tool and the workpiece is set as surface-to-surface contact in the explicit analysis because the contact surface between the tool and the workpiece continuously changes during the cutting process. To simulate the cutting scenario, the bottom and left sides of the workpiece are fixed. The cutting edge of the tool is in contact with the entire workpiece material. In the contact properties, both the tangential behavior and normal behavior of the tool are established. For the tangential behavior, the friction formulation is set to penalty with a friction coefficient of 0.5. The normal behavior contact pressure–overclosure relationship is set to “hard” contact. In this model, a triangular mesh is utilized to address the dynamic deformation characterized by particle shear, detachment, and plowing/extrusion processes, deviating from the conventional rectangular mesh approach [26]. A total of 43,542 triangular meshes were created from the partitioned workpiece. In this paper, when dividing triangular meshes, free meshing is used. The maximum angle does not exceed 120°, the minimum angle is not less than 30°, and the ratio of the longest edge to the shortest edge is between 1 and 2. To optimize the computational effectiveness, the mesh density is increased in the upper right section while remaining sparser in other areas. This mesh distribution is depicted in Figure 3a. Figure 3b depicts the workpiece and tool following assembly and highlights the cutting simulation area positioned in the upper right corner.

### 2.4. Experimental Validation and Data Analysis

**Figure 4:**
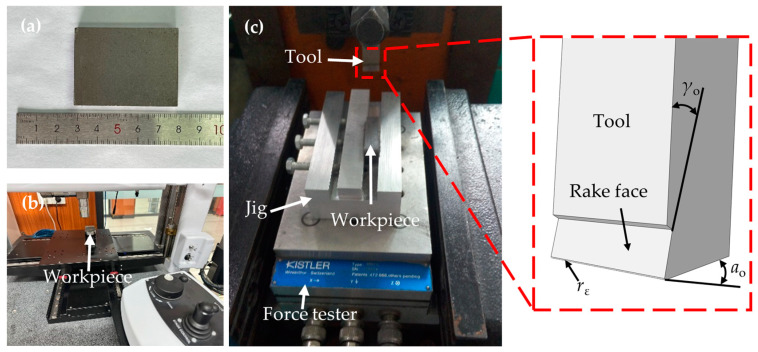
Experimental specimens and equipment. (**a**) PM green compacts specimen. (**b**) White light interferometer. (**c**) Cutting experiment on raw green compacts.

The simulation models were subjected to experimental verification to assess their accuracy. Figure 4a displays the PM green compact specimen used in this study. Multiple orthogonal cutting trials were conducted on these specimens, following which a white light interferometer, as shown in Figure 4b, was utilized to examine and measure the machined surface quality of the green compacts. The parameters employed during these cutting experiments were aligned as closely as possible with those of the simulation model, and these experiments are depicted in Figure 4c. 

**Figure 5:**
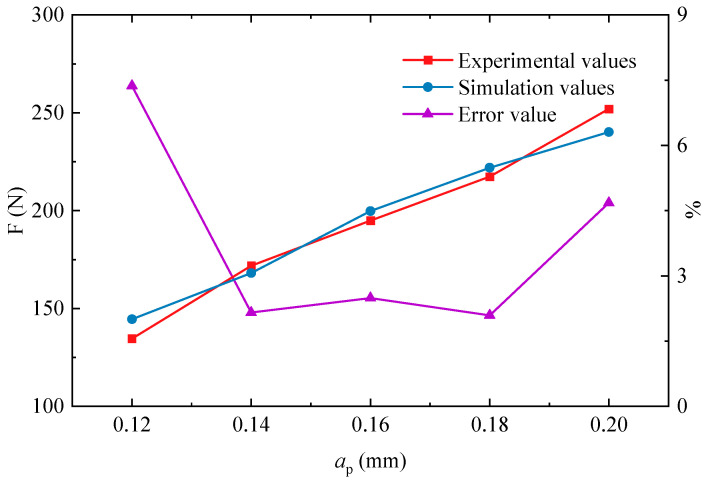
Cutting forces across various cutting thicknesses at a cutting speed of 5 m/min.

Figure 5 compares the simulated and experimental values of the cutting force exerted on the PM green compact specimens across different cutting thicknesses, with the cutting speed maintained at 5 m/min. Each value is the average of six experiments. For a cutting thickness of 0.12 mm, the discrepancy between the simulated value and the experimental value was 7.37%, while at other measured points, the variation did not exceed 5%, confirming the reliability of the model.

**Figure 6:**
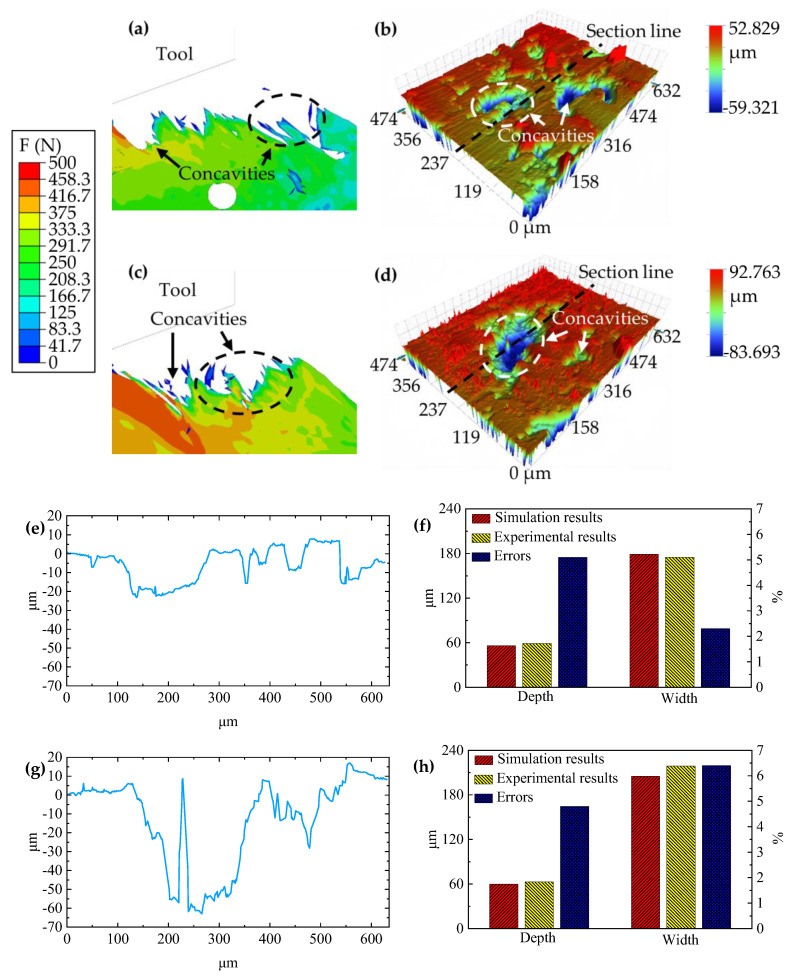
Analysis of machined surfaces through simulation and experimental approaches. (**a**) Simulated outcomes detailing the surface characteristics when employing a cutting speed of 65 m/min and a cutting thickness of 0.15 mm. (**b**) Corresponding experimental observations for a cutting regime characterized by a speed of 65 m/min and a thickness of 0.15 mm. (**c**) Simulation data for the surface generated at a cutting speed of 65 m/min, with the cutting thickness increased to 0.30 mm. (**d**) Experimental validation for the conditions set in panel c. (**e**) A detailed height profile of the machined surface for a cutting thickness of 0.15 mm. (**f**) A comparative analysis of the depth and width dimensions of the craters formed at a cutting thickness of 0.15 mm. (**g**) Height profile for the cutting thickness (0.35 mm). (**h**) Detailed comparison of crater dimensions—both depth and width—at a cutting thickness of 0.35 mm.

In this study, the evaluation criteria for surface quality are the size and depth of the concavities on the machined surface of the workpiece. During these tests, a constant speed of cutting of 65 m/min and a thickness of 0.15 mm were maintained. Figure 6a presents the simulation outcomes, while Figure 6b shows the corresponding experimental outcomes, with the left side of Figure 6e illustrating the height curve of the profile. The concavity depth in the simulation plot on the right side measures 56 μm with a width of 179 μm, whereas the experimental result on the left side shows a concavity depth of 59 μm and a width of 175 μm, yielding errors of 5.1% and 2.3%, respectively, as shown in Figure 6f. These results indicate that the simulation and experimental outcomes are closely matched, with the machined surface quality remaining satisfactory and the concavities exhibiting a small and shallow morphology. Figure 6c shows the simulation results for a cutting speed of 65 m/min and cutting thickness of 0.35 mm, and Figure 6d shows the experimental results, with the right side of Figure 6g showing the height curve of the profile. The concavity depth and width on the right side of the simulation plot are 60 μm and 205 μm, respectively. In contrast, the experimental results on the left side show a concavity depth of 63 μm and a width of 219 μm, with errors of 4.8% and 6.4%, respectively, as shown in Figure 6h. This comparison reveals that, while the simulation and experimental results generally align, the quality of the machined surface is inferior, characterized by a significant presence of burrs and large, deep concavities. In conclusion, the experimental evidence supports the validity of the model.

## 3. Results and Discussion

### 3.1. Analysis of the Cutting Process

**Figure 7:**
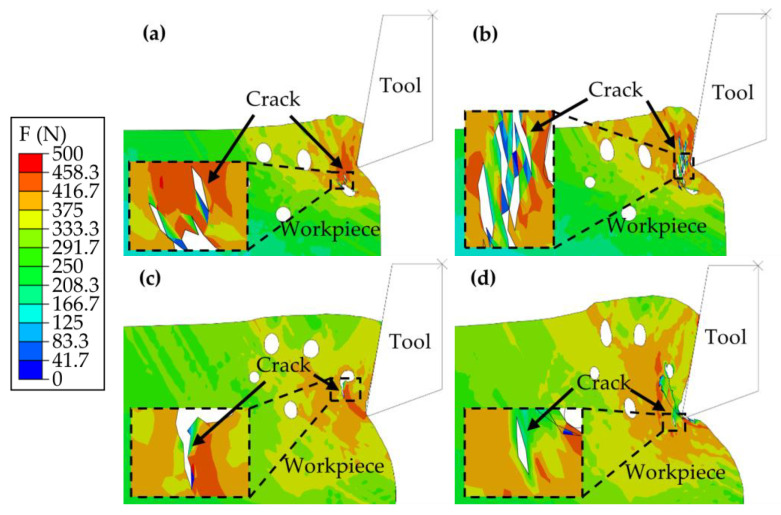
Initial phase of the cutting process. (**a**,**b**) Cutting at a thickness of 0.15 mm. (**c**,**d**) Cutting at a thickness of 0.35 mm.

The examination of the cutting process begins with its initial phase. Figure 7a,b illustrate the process for which the thickness of cutting was consistently 0.15 mm, whereas Figure 7c,d explore the process at a thickness of 0.35 mm.

Upon contact between the tool and the PM green compact specimen, the specimen at a cutting thickness of 0.15 mm exhibited cracking on the lower left side of the tool, as shown in Figure 7a, away from the specimen edge. Conversely, at a cutting thickness of 0.35 mm, cracking was observed on the upper left side of the tool, as illustrated in Figure 7c, which was also distant from the specimen edge. Notably, these cracks originated within the specimen, near the pores, diverging from typical crack initiation at the tool contact point. This unique pattern is attributed to the specimens being composed of unsintered metal powder compacts, which prevents them from behaving as monolithic entities. The stress concentration, which leads to crack formation at both cutting thicknesses, was located near the pores.

As the cutting advanced to the positions depicted in Figure 7b,d, the specimen with a cutting thickness of 0.15 mm exhibited further crack propagation and the emergence of numerous smaller cracks but without the formation of a machined surface. This phenomenon is attributed to the initial cracks being situated far from the specimen surface, which hinders their extension to the surface and causes them to propagate internally instead. In contrast, for the specimen with a thickness of 0.35 mm, cracks rapidly extended from the pore vicinity to the surface, culminating in the formation of a machined surface and additional cracks extending toward the lower left.

**Figure 8:**
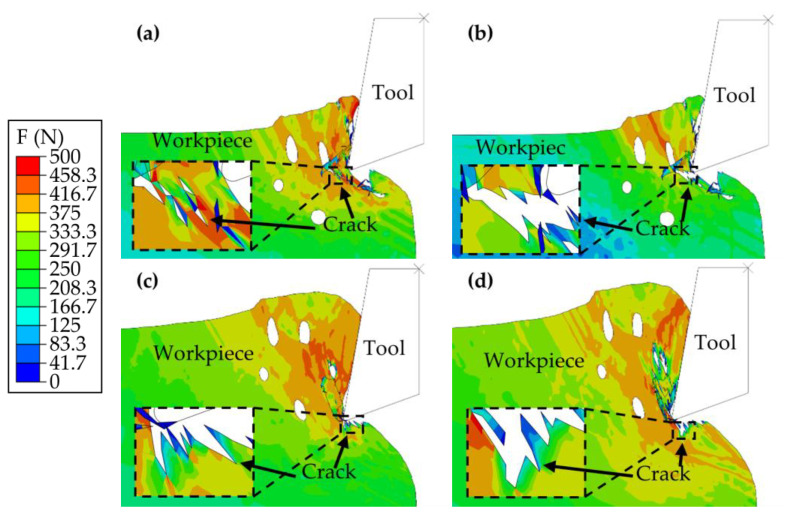
Mid-section analysis of the cutting process: (**a**,**b**) Cutting at a thickness of 0.15 mm; (**c**,**d**) Cutting at a thickness of 0.35 mm.

The analysis then progresses to the intermediate phase of the cutting process, with Figure 8a,b focusing on a cutting thickness of 0.15 mm and Figure 8c,d focusing on a thickness of 0.35 mm.

During the cutting phases depicted in Figure 8a,c, the machined surface on the specimen at a cutting thickness of 0.15 mm was formed, with a crack initiating within the specimen and propagating toward the left pore. Conversely, at a cutting thickness of 0.35 mm, the machined surface exhibited a crater originating from a preceding crack moving toward the lower left. As cutting proceeded to the stages shown in Figure 8b,d, the primary deformation zone in the 0.15 mm thick specimen continued to fracture, generating numerous small chips. This process resulted in a superior machined surface quality attributed to the absence of significant cracks on the machined surface. On the other hand, for the specimen with a cutting thickness of 0.35 mm, the previously formed cracks extended further downward, echoing the process outlined in Figure 7d. This process led to the formation of additional cracks extending toward the lower left, contributing to a rougher and inferior machined surface quality.

These findings underscore the profound impact of cutting thickness on the regularity of crack formation as well as the quality of the machined surfaces of the samples. It was determined that employing a smaller cutting thickness could diminish crack formation and enhance machined surface quality. In contrast, a greater cutting thickness is likely to facilitate crack propagation and increase surface roughness, thereby compromising the structural integrity and overall performance of the workpiece.

**Figure 9:**
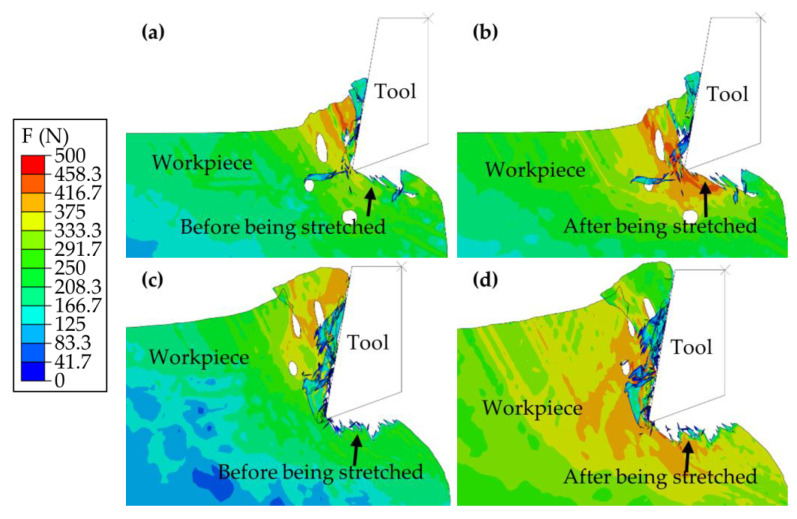
Latter stages of the cutting process (**a**,**b**) at a thickness of 0.15 mm and (**c**,**d**) at a thickness of 0.35 mm.

Subsequently, the analysis transitions to the latter stages of the cutting process. Figure 9a,b illustrate the cutting dynamics for a thickness of 0.15 mm, whereas Figure 9c,d depict the process for a thickness of 0.35 mm.

**Figure 10:**
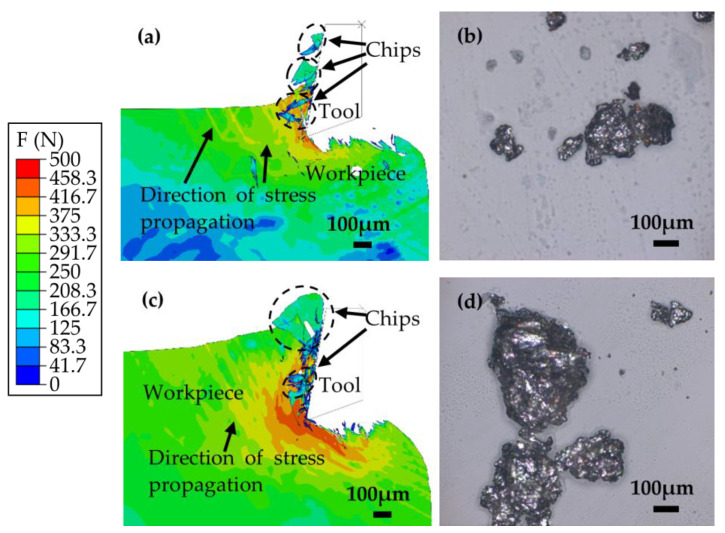
Analysis of specimen forces and chip formation during cutting. (**a**) Simulation of chip formation at a cutting thickness of 0.15 mm. (**b**) Experimental observation of chip formation at a cutting thickness of 0.15 mm. (**c**) Simulation of chip formation at a cutting thickness of 0.35 mm. (**d**) Experimental observation of chip formation at a cutting thickness of 0.35 mm.

As the cutting process progresses beyond the stages illustrated in Figure 7 and Figure 8, the previously observed patterns of crack formation cease to apply. Over time, the specimens cease to develop new cracks, as depicted in Figure 9a,c. Subsequently, the machined surfaces undergo stretching, which causes a decrease in surface quality, as shown in Figure 9b,d. After a certain duration, the specimens develop a new crack, reverting to the conditions observed in Figure 7a,c, thereby initiating the next cycle. This observation underscores the way in which, despite being classified as a brittle material, PM green compacts exhibit a deformation stage that does not fully conform to the brittle material cutting model. As demonstrated in Figure 9, the deformation stage in specimens with a 0.15 mm cutting thickness is less pronounced than that in specimens with a cutting thickness of 0.35 mm. The prolonged deformation stage in the latter results in significant concavity on the machined surface, culminating in the formation of a large, deep crater that adversely impacts surface quality. Figure 10 further elucidates this phenomenon. Figure 10a,b present the simulation and experimental findings for chip formation at a cutting thickness of 0.15 mm, respectively, while Figure 10c,d showcase the corresponding outcomes at a cutting thickness of 0.35 mm.

Figure 10a shows that the specimen subjected to a cutting thickness of 0.15 mm exhibits a distinct direction of stress propagation due to the action of the tool. The stress predominantly affects the upper part of the specimen, manifesting in a linear and parallel orientation. This force distribution facilitates the formation of chips, which are neither excessively large nor small, and significantly reduces the cutting force and the deformation phase of the specimen. Conversely, the stress pattern in the specimen with a cutting thickness of 0.35 mm is less defined, resulting in increased cutting force, an extended deformation phase, and a compromised machined surface quality, as illustrated in Figure 10c.

At a cutting thickness of 0.15 mm, the chips produced have a more consistent size and shape and are devoid of oversized or undersized anomalies. Similarly, this thickness maintains the regularity in chip size and shape, preventing any excessively large or small chips. At a thickness of 0.35 mm, however, the chips display considerable variation in size and shape, with a tendency toward larger dimensions accompanied by numerous smaller fragments. The congruence between the simulation results and the experimental results lends further credence to the accuracy of the model.

### 3.2. Significance Analysis of the Cutting Force Factors

Orthogonal testing, which is a strategic experimental design method, is employed to systematically scrutinize the influence of various factors on a system or process. The aim of orthogonal testing is to identify the most favorable process conditions, parameter configurations, or design solutions by gaining a thorough understanding of these factors and their interplay while minimizing the number of experiments needed. To enrich the dataset, a 4-factor, 5-level orthogonal test was devised. Parameters such as the cutting thickness (*a*_p_), cutting speed (*v*_c_), tool rake angle (*γ*_o_), and radius of the rounded edge (*r*_ε_) were central to the study.

The cutting thickness (*a*_p_) levels ranged from 0.15 mm to 0.35 mm and were chosen to capture a broad spectrum of practical applications. The cutting speed (*v*_c_) levels varied from 5 m/min to 65 m/min, reflecting common machining conditions. Tool rake angle (*γ*_o_) levels were selected based on typical tool geometries used in machining PM green compacts, and the radius of the rounded edge (*r*_ε_) levels were chosen to study the effect of tool edge sharpness.

Analysis of variance is widely used as a statistical method to assess the mean differences across distinct groups. The four-factor ANOVA represents an extended application of ANOVA and is tailored for studies involving four independent variables. The integration of orthogonal tests with ANOVA facilitates a more detailed and systematic exploration of the impacts of multiple factors on a system, thus providing deeper insight into system optimization [31,32,33,34]. The subsequent section will outline the computation process of the four-factor ANOVA employed in this study, including the determination of main effects and interactions. This approach is aimed at providing a more transparent interpretation of the test outcomes. The detailed simulation data are listed in Table 4.

The methodology for computing the data in Table 4 is delineated below [34,35]:

Calculate the total sum of squares (*SS*_T_):

(1) $${\mathrm{SS}}_{T}=\sum_{i=1}^{n}\sum_{j=1}^{k}y_{\mathrm{ij}}^{2}-\mathrm{CT}$$

where *y*_ij_ denotes the cutting force for each set of simulations, and CT is the correction factor:

(2) $$\mathrm{CT}=\frac{{\left(\sum_{i=1}^{n}\sum_{j=1}^{k}y_{ij}\right)}^{2}}{\mathrm{nk}}$$

Calculate the sum of squares for each factor (*SS*_F_) and the error (*SS*_E_):

(3) $${\mathrm{SS}}_{F}=\frac{\sum {\left(\mathrm{factor}\;\mathrm{sum}\right)}^{2}}{\mathrm{number}\;\mathrm{of}\;\mathrm{levels}}-\mathrm{CT}$$

(4) $${\mathrm{SS}}_{E}={\mathrm{SS}}_{T}-\sum {SS}_{F}$$

Determine the degrees of freedom (DF), mean square (MS), and *F* value:

(5) DF=number of levels−1

(6) $$\mathrm{MS}=\frac{\mathrm{SS}}{\mathrm{DF}}$$

(7) $$F=\frac{{\mathrm{MS}}_{F}}{{\mathrm{MS}}_{E}}$$

In Table 5, the terms “**”, “ns”, and “/” indicate highly significant effects, nonsignificant effects, and not applicable, respectively. The critical values *F*_0.05_ and *F*_0.01_ are obtained from *F* distribution tables and represent the threshold values at the 5% and 1% significance levels, respectively. These values are used to determine whether the observed *F* values indicate a statistically significant effect of the factors on the response variable.

Table 5 presents the ANOVA results for the cutting force. The analysis reveals that the cutting thickness (*a*_p_) significantly impacts the cutting force, with an *F* value of 177.781, which is much higher than the critical values *F*_0.05_ = 3.84 and *F*_0.01_ = 7.01, indicating a highly significant effect. Conversely, the cutting speed (*v*_c_), tool rake angle (*γ*_o_), and radius of the rounded edge (*r*_ε_) have *F* values of 0.125, 2.457, and 1.620, respectively, which are all lower than the critical value *F*_0.05_. This suggests that these factors do not have a statistically significant impact on the cutting force.

### 3.3. Optimization of the Cutting Force Parameters

The orthogonal experimental ANOVA that was previously discussed enabled the identification of the influence of various parameters on system performance through a structured experimental design and data analysis approach. Nevertheless, for complex issues, conventional experimental designs and statistical methods might encounter difficulties due to vast parameter spaces, nonlinear relationships, or multimodality.

**Figure 11:**
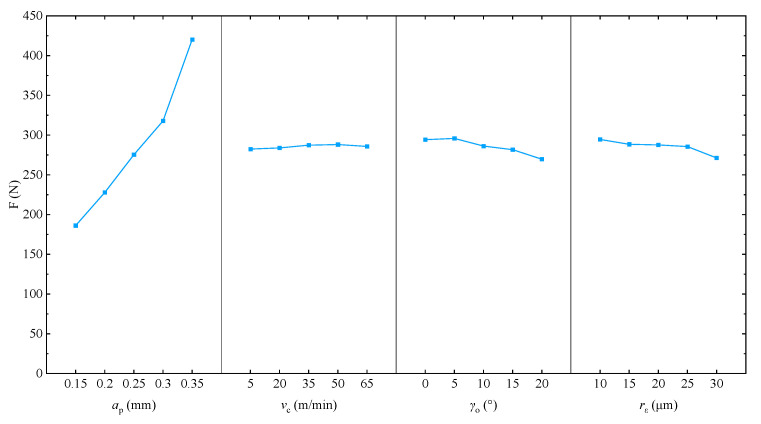
Trend of mean-level k for each factor.

This study considers four principal parameters for cutting, namely, the cutting thickness (*a*_p_), cutting speed (*v*_c_), tool rake angle (*γ*_o_) and radius of the rounded edge (*r*_ε_), as well as their respective cutting force values. Figure 11 depicts the trend of the mean level k for each parameter. 

While cutting thickness significantly influences cutting force, developing algorithms to determine cutting forces remains essential since cutting force is also affected by other parameters such as cutting speed, tool rake angle, and radius of the rounded edge. The interactions among these factors necessitate more sophisticated models for accurate prediction. Optimization algorithms can enhance prediction accuracy and balance multiple objectives, such as minimizing cutting force while maximizing surface quality and production efficiency. Therefore, developing these algorithms is crucial for improving the accuracy and efficiency of the cutting process and provides valuable references for related research.

To comprehensively assess the influence of these four factors on cutting force and to enhance optimization, this study adopts the GANN, an advanced optimization method [36,37,38].

Genetic algorithms (GAs) are optimization techniques that emulate natural selection and genetic processes, using simulations of genetic inheritance, mutation, and selection to iteratively refine model parameters, thereby attaining an optimal solution. In this context, GAs adjust the weights and biases within the neural network to better represent the intricate relationships between cutting forces and their parameters.

Neural networks, which are known for their robust nonlinear modeling capability, excel at fitting complex input–output relationships. However, their training requires extensive data and parameter adjustments, with a tendency to converge to local optima. To mitigate these issues, this research integrates the global search capability of genetic algorithms with the fitting ability of the neural network, establishing an effective optimization framework for cutting force optimization challenges. MATLAB (2021a) software will be employed to develop and execute the genetic algorithm program.

In this study, data on cutting parameters—cutting speed, tool rake angle, and radius of the rounded edge—and corresponding cutting force measurements were collected. Subsequently, a feedforward neural network with input, hidden, and output layers was designed.

In this research, the weights and biases of the neural network serve as the optimization variables for the genetic algorithm to minimize the prediction error of the neural network. These parameters are encoded as individuals within the genetic algorithm, which then undergoes evolutionary optimization until an optimal set of weights and biases is identified. To assess the performance of the neural network model, a fitness function is defined, which calculates the prediction error to serve as a criterion for evaluating the genetic algorithm.

The number of input layer nodes in the neural network depends on the number of cutting parameters, with one output layer node. There are two hidden layers with sizes of 10 and 8. The weight and bias parameters for the genetic algorithm have upper and lower bounds of 1 and −1, with a maximum generation number of 100 and a population size of 50. Through these processes, the integration of the genetic algorithm with the neural network for cutting force optimization is effectively realized.

Optimal results were achieved with a cutting thickness of 0.15 mm, a cutting speed of 20 m/min, a tool rake angle of 10°, and a radius of the rounded edge of 25 μm, leading to a cutting force of 174.998 N. When these parameters are simulated in the model, the resultant cutting force is 168.189 N, which indicates an error of 4.05%. This result underscores the accuracy and success of cutting force optimization.

## 4. Conclusions

In this article, Abaqus (2022) software facilitated the modeling of PM green compacts, followed by an analysis of the simulation results using orthogonal test ANOVA and an advanced analysis utilizing MATLAB (2021a) software in conjunction with the GANN. The principal findings are summarized as follows:

(1) A refined model of PM compacts was developed, yielding an average cutting force error of 3.8% within a cutting thickness range of 0.12–0.20 mm. Additionally, the average errors for the concavity depth and width on the machined surface were 5.0% and 4.4%, respectively.
(2) PM green compacts, characterized as brittle materials, exhibit plastic deformation during cutting, deviating from the traditional cutting model for brittle materials. This observation offers fresh perspectives on cutting PM green compacts, thus improving the understanding of their machining dynamics.
(3) The cutting thickness has the most substantial impact on the cutting force, while the speed of cutting, the tool rake angle, and the radius of the rounded edge have minimal effects. This finding underscores the importance of cutting thickness control in PM green compact machining to prevent damage due to excessive cutting force.
(4) The optimization of the neural network using genetic algorithms determined the ideal parameter set for cutting PM green compacts, as follows: a cutting thickness of 0.15 mm, a cutting speed of 20 m/min, a tool rake angle of 10°, and a radius of the rounded edge of 25 μm. This parameter set led to a cutting force of 174.998 N with a 4.05% deviation from the actual measurement, which provides a valuable reference for machining PM green compacts.

## Figures and Tables

**Table 1:** Mechanical characteristics of green compact materials constructed via powder metallurgy.

Performance	Density (g/cm^3^)	Vickers Hardness (HV)	Tensile Strength (MPa)	Compressive Strength (MPa)	Elastic Modulus (GPa)	Poisson’s Ratio
Parameter	7.1	87	3.9	98	210	0.018

**Table 2:** Chemical composition of the green compact material.

Chemical Composition	Fe	C	O	S	Mn	Mo	Ni	Cu
Proportion (%)	96.0586	0.002	0.07	0.0074	0.136	0.506	1.75	1.47

**Table 3:** Variables for PM green compacts in the Johnson–Cook constitutive algorithm.

Parameter	*A* (MPa)	*B* (MPa)	*C*	*m*	*n*	*T*m (°C)	*T*r (°C)
Value	101	91	0.127	1.46	0.213	1861	25

**Table 4:** Simulation data for the cutting force.

Test Number (i)	Four Factors				*F*_H_ (*y*_i_)
	*a*_p_ (mm)	*v*_c_ (m/min)	*γ*_o_ (°)	*r*_ε_ (μm)	
1	0.15	5	0	10	183.564
2	0.15	20	10	25	184.028
3	0.15	35	20	15	186.512
4	0.15	50	5	30	189.603
5	0.15	65	15	20	186.964
6	0.2	5	20	25	212.920
7	0.2	20	5	15	230.748
8	0.2	35	15	30	212.350
9	0.2	50	0	20	237.716
10	0.2	65	10	10	245.292
11	0.25	5	15	15	263.694
12	0.25	20	0	30	275.158
13	0.25	35	10	20	273.018
14	0.25	50	20	10	280.353
15	0.25	65	5	25	284.094
16	0.3	5	10	30	308.031
17	0.3	20	20	20	296.932
18	0.3	35	5	10	330.820
19	0.3	50	15	25	312.766
20	0.3	65	0	15	340.883
21	0.35	5	5	20	443.402
22	0.35	20	15	10	432.286
23	0.35	35	0	25	433.532
24	0.35	50	10	15	420.143
25	0.35	65	20	30	371.171

**Table 5:** ANOVA results for the cutting force.

Variation Source	Square of Deviance	Degree of Freedom	Sum of Mean Squares	*F*	Significance	*F*_0.05_	*F*_0.01_
*a*_p_ (mm)	162,374.456	4	40,593.614	177.781	**	3.84	7.01
*v*_c_ (m/min)	113.973	4	28.493	0.125	ns		
*γ*_o_ (°)	2244.428	4	561.107	2.457	ns		
*r*_ε_ (μm)	1479.226	4	369.807	1.620	ns		
Error	1826.676	8	228.335	/	/		
Summation	168,038.759	24	/	/	/		

## Data Availability

All data to support the results of this study are included in this article.

## References

[1] Zeng K., Wu X., Jiang F., Shen J.Y., Zhu L.F., Li L. (2023). A comprehensive review on the cutting and abrasive machining of cemented carbide materials. J. Manuf. Process..

[2] Du J.G., Tian B., Duan L.Y., Ming W.Y., Liu K., He W.B. (2022). Traditional and non-traditional machining technology of metallic glass. Int. J. Adv. Manuf. Technol..

[3] Li Y., Zhang X.H., Hao J.Y., Huang Q.B. (2022). The impact of green technology innovation on global value chain upgrading in China’s equipment manufacturing industry. Front. Environ. Sci..

[4] Zhu J.H., Zhou H., Wang C., Zhou L., Yuan S.Q., Zhang W.H. (2021). A review of topology optimization for additive manufacturing: Status and challenges. Chin. J. Aeronaut..

[5] Erdem M., Cinici H., Gokmen U., Karakoc H., Turker M. (2016). Mechanical and ballistic properties of powder metal 7039 aluminium alloy joined by friction stir welding. Trans. Nonferrous Met. Soc. China.

[6] Tijo D., Kumari S., Masanta M. (2017). Hard and wear resistance TiC-composite coating on AISI 1020 steel using powder metallurgy tool by electro-discharge coating process. J. Braz. Soc. Mech. Sci. Eng..

[7] Zharov M.V. (2023). Comparative Analysis of Features of Technologies for Obtaining Quality Spherical Powder of Nickel Aluminide NiAl. Metallurgist.

[8] Boujelbene M., Ezeddini S., Ben Said L., Bayraktar E., Alhadri M., Aich W., Ghachem K., Kolsi L. (2023). Analysis of surface integrity of intermetallic composite based on titanium-aluminum machined by laser cutting. Opt. Laser Technol..

[9] Egorov M.S., Egorova R.V. (2023). Characteristics of the Formation of Structure and Properties of Dispersed-Hardened Charges at the Hot Stamping of Complex Shape Products. Metallurgist.

[10] Zhong B., Wang Y.R., Wei D.S., Zhang K.S., Wang J.L. (2018). Multiaxial fatigue life prediction for powder metallurgy superalloy FGH96 based on stress gradient effect. Int. J. Fatigue.

[11] Sun F.J., Qu S.G., Li G., Pan Y.X., Li X.Q. (2016). Comparison of the machinability of titanium alloy forging and powder metallurgy materials. Int. J. Adv. Des. Manuf. Technol..

[12] da Silva G.C., Saraiva Q.M., Carneiro J.R.G., Santos I.A., José A.S. (2021). New milling methodology for sealing planes in AlSi9Cu3(Fe) alloy machined with PCD tool. Int. J. Adv. Manuf. Technol..

[13] Dong X.J., Wu J.Q., Yu H.L., Zhou Q., Wang W.Q., Zhang X.Q., Zhang L., Li L., He R.J. (2022). Additive manufacturing of silicon nitride ceramics: A review of advances and perspectives. Int. J. Appl. Ceram. Technol..

[14] Yin Y.K., Xu J.Y., Ji M., Li L.F., Chen M. (2023). A critical review on sintering and mechanical processing of 3Y-TZP ceramics. Ceram. Int..

[15] Attia U.M. (2021). Cold-isostatic pressing of metal powders: A review of the technology and recent developments. Crit. Rev. Solid State Mater. Sci..

[16] Kulkarni H., Dabhade V. (2019). Green machining of powder-metallurgy-steels (PMS): An overview. J. Manuf. Process..

[17] Chen C., Huang B.Y., Liu Z.M., Li Y.X., Zou D., Liu T., Chang Y.M., Chen L. (2023). Additive manufacturing of WC-Co cemented carbides: Process, microstructure, and mechanical properties. Addit. Manuf..

[18] Parvizi S., Hashemi S.M., Asgarinia F., Nematollahi M., Elahinia M. (2021). Effective parameters on the final properties of NiTi-based alloys manufactured by powder metallurgy methods: A review. Prog. Mater. Sci..

[19] Bram M., Ebel T., Wolff M., Cysne Barbosa A.P., Tuncer N. (2013). Applications of Powder Metallurgy in Biomaterials. Advances in Powder Metallurgy.

[20] Paradis L., Waryoba D., Robertson K., Ndayishimiye A., Fan Z.M., Rajagopalan R., Randall C.A. (2022). Densification and Strengthening of Ferrous-Based Powder Compacts Through Cold Sintering Aided Warm Compaction. Adv. Eng. Mater..

[21] Robert-Perron E., Blais C., Pelletier S. (2009). Tensile properties of sinter hardened powder metallurgy components machined in their green state. Powder Metall..

[22] Yang D.Y., Lu L.S., Wan Z.P. (2020). Material Removal Mechanism of Green Machining on Powder Metallurgy Parts during Orthogonal Cutting. Adv. Mater. Sci. Eng..

[23] Shi Q.L., Chen D., Pan Q.Q., Peng J.G. (2023). Study on the Influence of Warm Die Compaction Behavior on the Mechanical Properties of Iron-Based Powder Materials. J. Mater. Eng. Perform..

[24] Kulkarni H., Dabhade V.V., Blais C. (2023). Analysis of machining green compacts of a sinter-hardenable powder metallurgy steel: A perspective of material removal mechanism. CIRP J. Manuf. Sci. Technol..

[25] Goncalves M., Sanchez L.E.D., Franca T.V., Fortulan C.A., da Silva R.H.L., Foschini C.R. (2019). Analysis of the tool nose radius influence in the machining of a green ceramic material. Int. J. Adv. Manuf. Technol..

[26] Kumar P., Karsh P.K., Misra J.P., Kumar J. (2021). Multi-objective optimization of machining parameters during green machining of aerospace grade titanium alloy using Grey-Taguchi approach. Proc. Inst. Mech. Eng. Part E.

[27] Yang D.Y., Zhang Y.C., Wang R., Wei F.R., Zeng L.X., Liu M. (2023). Finite Element Modeling and Optimization Analysis of Cutting Force in Powder Metallurgy Green Compacts. Processes.

[28] Bidulska J., Bidulsky R., Grande M.A., Kvackaj T. (2019). Different Formation Routes of Pore Structure in Aluminum Powder Metallurgy Alloy. Materials.

[29] Shokry A. (2019). On the constitutive modeling of a powder metallurgy nanoquasicrystalline Al_93_Fe_3_Cr_2_Ti_2_ alloy at elevated temperatures. J. Braz. Soc. Mech. Sci..

[30] Sun Y., Li G.H., He Z., Kong X.C. (2022). The advance of research on constitutive model used in finite element simulation of metal cutting. Proc. Inst. Mech. Eng. C J. Mech. Eng. Sci..

[31] Hüseyin G., Yunus E.G. (2020). Optimization and evaluation of dry and minimum quantity lubricating methods on machinability of AISI 4140 using Taguchi design and ANOVA. Proc. Inst. Mech. Eng. Part C J. Mech. Eng. Sci..

[32] Naik A.B., Reddy A.C. (2018). Optimization of Tensile Strength in TIG Welding Using Taguchi Method and Analysis of Variance (ANOVA). Therm. Sci. Eng. Prog..

[33] Savković B., Kovăc P., Stoić A., Dudić B. (2020). Optimization of Machining Parameters Using the Taguchi and ANOVA Analysis in the Face Milling of Aluminum Alloys AL7075. Teh. Vjesn..

[34] Deng C.S., Hsia K.L. (2017). Analysis of the optimisation of parameters for the laser machining of timber. Int. J. Adv. Manuf. Technol..

[35] Kruzel R., Dembiczak T., Wachowicz J. (2023). Optimization of Spark Plasma Sintering Technology by Taguchi Method in the Production of a Wide Range of Materials: Review. Materials.

[36] Elkatatny S., Alsharekh M.F., Alateyah A.I., El-Sanabary S., Nassef A., Alawad M.O., Kamel M., BaQais A., El-Garaihy W.H., Kouta H. (2023). Optimizing the Powder Metallurgy Parameters to Enhance the Mechanical Properties of Al-4Cu/xAl_2_O_3_ Composites Using Machine Learning and Response Surface Approaches. Appl. Sci..

[37] Wen H.N., Jin J.S., Tang X.F., Wang X.Y., Yang H.Y., Zhang Y.D., Zhang M., Deng L., Wei Q.S., Chen J.Z. (2023). Machine learning-assisted constitutive modeling of a novel powder metallurgy superalloy. Int. J. Mech. Sci..

[38] Dreano A., Favre J., Desrayaud C., Chanin-Lambert P., Wimmer A., Zaeh M.F. (2022). Computational design of a crack-free aluminum alloy for additive manufacturing br. Addit. Manuf..

